# Raman imaging as a new approach to identification of the mayenite group minerals

**DOI:** 10.1038/s41598-018-31809-4

**Published:** 2018-09-11

**Authors:** D. Środek, M. Dulski, I. Galuskina

**Affiliations:** 1 Faculty of Earth Sciences, Department of Geochemistry, Mineralogy and Petrography, University of Silesia, Bedzińska 60, 41-200 Sosnowiec, Poland; 2 Institute of Material Science, University of Silesia, Uniwersytecka 4, 40-007 Katowice, Poland; 3 Silesian Center for Education and Interdisciplinary Research, 75 Pułku Piechoty 1a, 41-500 Chorzów, Poland

## Abstract

The mayenite group includes minerals with common formula Ca_12_Al_14_O_32−x_(OH)_3x_[*W*_6−3x_], where *W* = F, Cl, OH, H_2_O and x = 0–2. This distinction in the composition is associated with *W* site which may remain unoccupied or be occupied by negatively charged ions: OH^−^, F^−^, Cl^−^, as well as neutral molecules like H_2_O. However, there is no experimental approach to easily detect or differentiate mineral species within the mayenite group. Electron micro-beam facilities with energy- or wavelength-dispersive X-ray detectors, as most common tools in mineralogy, appear to be insufficient and do not provide a definite identification, especially, of hydroxylated or hydrated phases. Some solution provides typical Raman analysis ensuring identification of minerals and 3D Raman imaging as an innovative approach to distinguish various co-existing minerals of the mayenite group within a small area of the rock sample. Raman spectroscopy has also been successfully used for a determination of water type incorporated into the mineral structure as well as for a spatial distribution of phases by cluster approach analysis and/or integrated intensity analysis of bands in the hydroxyl region. In this study, Raman technique was for the first time used to reconstruct a 3D model of mayenite group mineral zonation, as well as to determine a way of water incorporation in the structure of these minerals. Moreover, for the first time, Raman data were correlated with alterations during the mineral-forming processes and used for reconstruction of the thermal history of studied rock. As a result, the influence of combustion gases has been proposed as a crucial factor responsible for the transformation between fluormayenite and fluorkyuygenite.

## Introduction

The ideal phase called “mayenite”, with the crystal chemical formula Ca_12_Al_14_O_33_ was synthesized only in laboratory conditions so far, and it is widely used in the ceramic industry or as an anion conductor^1^. However, in nature, a phase with such chemical composition has not been noted, as yet. The name “mayenite” has been first used by Hentschel in 1964, who described mineral with a composition similar to synthetic phase from calcium-silicate xenolith enclosed in basalt volcanic rocks from Bellerberg, Eifel, Germany. Unfortunately, the mineral composition was determined incorrectly^2^. In last decade, a re-investigation of holotype specimen from Eifel provided an opportunity to redefine and rename this mineral as chlormayenite with a crystal chemical formula of the end-member Ca_12_Al_14_O_32_[▯_4_Cl_2_], where ▯ means vacancy^3,4^. Description of a few new mineral species related to chlormayenite recently demanded an elaboration of a new classification of the mayenite supergroup, which combines two isostructural mineral groups – wadalite group (silicates) and mayenite group (oxides)^4^. The general crystal chemical formula of the mayenite supergroup minerals is as follows: X_12_T_14_O_32−x_(OH)_3x_[*W*_6−3x_], where x–polyhedral site occupied by Ca; T – tetrahedral site occupied by Al^3+^, Fe^3+^, Mg^2+^, Si^4+^,Ti^4+^; *W* site in the centre of structural cages occupied by OH^−^, F^−^, Cl^−^ as well as neutral molecules like H_2_O, 0 ≤ x < 0.75^3–7^. In synthetic mayenites *W* site can be occupied by e^−^, O_2_^−^, O_2_^2−^,O^−^, S^2−^, OH^−^, N_x_^−^, CN^−^, F^−^, Cl^−^, Au− ^8–15^. The porous nature of the mayenite enforces its economy meaning as a potential water clathrate or modifier of Portland cement^16–18^. This is due to the tendency of its relatively easy hydration as a result of replacing oxygen by hydroxyl group or water molecules^3,5,7,19,20^. However, the presence of H_2_O was described only in natural phases^5,7^.

Fluormayenite Ca_12_Al_14_O_32_[▯_4_F_2_] and fluorkyuygenite Ca_12_Al_14_O_32_[(H_2_O)_4_F_2_] are characterised by physical features, atom arrangement and crystal structure similar to other members of the mayenite group, previously reported in literature^4,5^. There are usually colorless, with transparent or vitreous lustre, and conchoidal or irregular fracture. Its crystallize as isometric rounded grains up to 50 μm in size. There are isotropic, sometimes with optical anomalous anisotropy and a refractive index close to 1.61. The temperature of fluormayenite formation is more than 800 °C, whereas fluormayenite to fluorkyuygenite transformation comes at a temperature about 400 °C^5^. Structure of fluormayenite and fluorkyuygenite is represented by tetrahedral framework {T_14_O_32_} with negative charge −22, which forms six structural cases coordinated by two Ca^2+^ with charge +24^21^. A surplus of positive charge equal +2 is compensated by two F^−^ at the *W* site^4^. Occupation of the *W* site is the main difference between fluormayenite and fluorkyuygenite structures. In structural cages of fluorkyuygenite, up to 4 molecules of water can be located beside two F^−^, whereas in fluormayenite only two from the six structural cages are occupied by fluorine while the others are empty^5^. A small amount of fluorine in the *W* site can be replaced by OH groups^5^. Moreover, additional OH groups (not in central *W* site), connected with changing of Al polyhedra coordination from tetrahedral to octahedral^5^, can be located in these structural cages, forming their walls.

The identification of mayenite-type minerals, using electron micro-beam apparatus with energy- (EDS) or wavelength-dispersive (WDS) X-ray detectors, is difficult and uncertain due to different types of water which may incorporate in crystal structure as hydroxyl group^4–6^, “zeolitic” or absorbed water^3,7^. The newest studies reported that water in minerals of mayenite group can also be presented in form of hydrogarnet defects^21^. Presence of different types of water in mayenites makes impossible to different members of this group using only chemical analysis. One of the solutions seems to be the application of structurally-sensitive Raman spectroscopy, which improves the quality of the analysis by distinguishing bands related to different water types. As a result, this technique might be useful in the distinction of fluormayenite and fluorkyuygenite. Moreover, integrated intensity and/or cluster analysis give an opportunity to follow a variability of H_2_O distribution within individual grains in the host rock. More detailed studies have indicated that seemingly homogeneous fluormayenite crystals show zonation related to the presence of various types of water in structure, similar to other mineral reported in literature such as chlorkyuygenite rim around wadalite core^7^. Such observations seem to be important in terms of rock genesis and petrological processes. In addition, due to the high mobility of anions, minerals of the mayenite group can be indicators of the combustion gases and fluids present in mineral-forming system^7^. In this context, fluormayenite might be considered as a primary rock-forming mineral, which further transforms to fluorkyugenite at the later stages accompanied by water vapor effect^5^.

Therefore, the main goal of this paper is the analysis of chemical composition and crystal structure allowing to specify the minerals of the mayenite group. The greatest emphasis was placed on the distinction of minerals with various degree of hydration and development of a genetic model of water incorporation into fluormayenite-type structure. In this purpose, the new approach based on the 3D Raman imaging was applied to follow spatial differentiation and establish a model of fluormayenite to fluorkyuygenite transformation.

## Occurrence and Paragenesis

The minerals of the mayenite group occur in pyrometamorphic larnite rock at Nahal Darga locality at the Judean Desert in Palestine^22^. Nahal Darga belongs to the Hatrurim Complex - a geological formation extending into the Israel, Palestine, and Jordan. There are two main hypotheses explained formation of pyrometamorphic rocks of the Hatrurim Complex related to high-temperature alteration of sedimentary protolith as a result of natural burning processes (1) of dispersed organic matter contained in primary sedimentary rocks^23–25^ and (2) high-temperature pyrometamorphism of primary rocks driven by methane combustion exhaling from tectonic active zones of the Dead Sea rift^22,26^. Larnite pseudoconglomerates are one of the most interesting rocks of the Hatrurim Complex. They consist of pebbles, showing large variety of mineral composition. Also within the individual pebbles a distinct zonation can be visible. It is caused by diversity of petrological processes, often with very restricted impact range, what resulted in crystallization of unusual minerals, such as shulamitite Ca_3_TiFe^3+^AlO_8_^27^; vapnikite Ca_2_CaUO_6_^28^; harmunite CaFe_2_O_4_^29^ or nabimusaite KCa_12_(SiO_4_)_4_(SO_4_)_2_O_2_F^30^.

**Figure 1:**
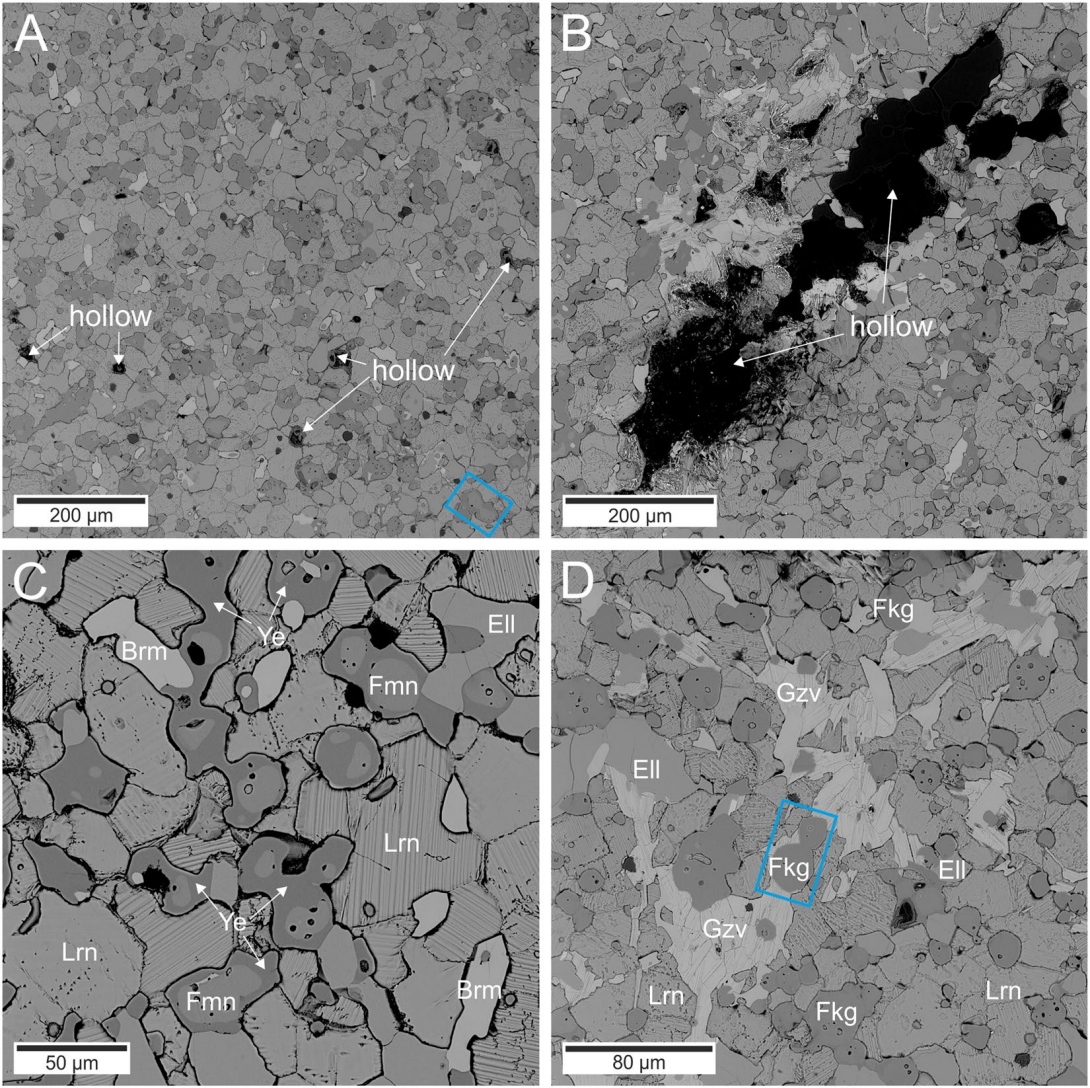
Backscattered electron image of fluormayenite- and fluorkyuygenite-bearing zones. (**A**) Porosity of zone with fluormayenite. (**B**) Porosity of zone with fluorkyuygenite. (**C**) Fluormayenite crystals with ye’elimite rims. (**D**) Typical fluorkyuygenite crystals. Brm - brownmillerite; Ell – fluorellestadite, Fkg – fluorkyuygenite, Fmn – fluormayenite, Gzv -gazeevite, Lrn – larnite, Ye – ye’elimite. Grains, for which Raman mapping was performed, are shown in the blue rectangles.

Nahal Darga locality generally consists of two areas (both about 5 km^2^) including unusual high-temperature (800–1250 °C) and low-pressure pyrometamorphic mineral associations. The studied samples were found in a small outcrop containing pseudo-conglomerate located about 1–1.5 km to the north from canyon Darga. The rock samples were brown in colour, and found as rounded pebbles consisting of larnite, mainly. The size of pebbles changes from few centimetres up to 50 cm in size. Larnite, β-Ca_2_SiO_4_, fluorellestadite, Ca_5_(SiO_4_)_1.5_(SO_4_)_1.5_F, brownmillerite, Ca_2_Fe^3+^AlO_5_, ye’elimite Ca_4_Al_6_O_12_SO_4_ and perovskite, CaTiO_3_ are the main minerals. Additionally, among the rock-forming minerals, two fluorine members of the mayenite group: fluormayenite as a common phase in low-porosity massive part of pebble (Fig. 1A), and fluorkyuygenite, which is distributed only within porous linear zones, were distinguished (Fig. 1B). Fluorkyuygenite (Fig. 1D) in BSE image is notably darker than fluormayenite (Fig. 1C) and it is devoid of ye’elimite rims.

**Figure 2:**
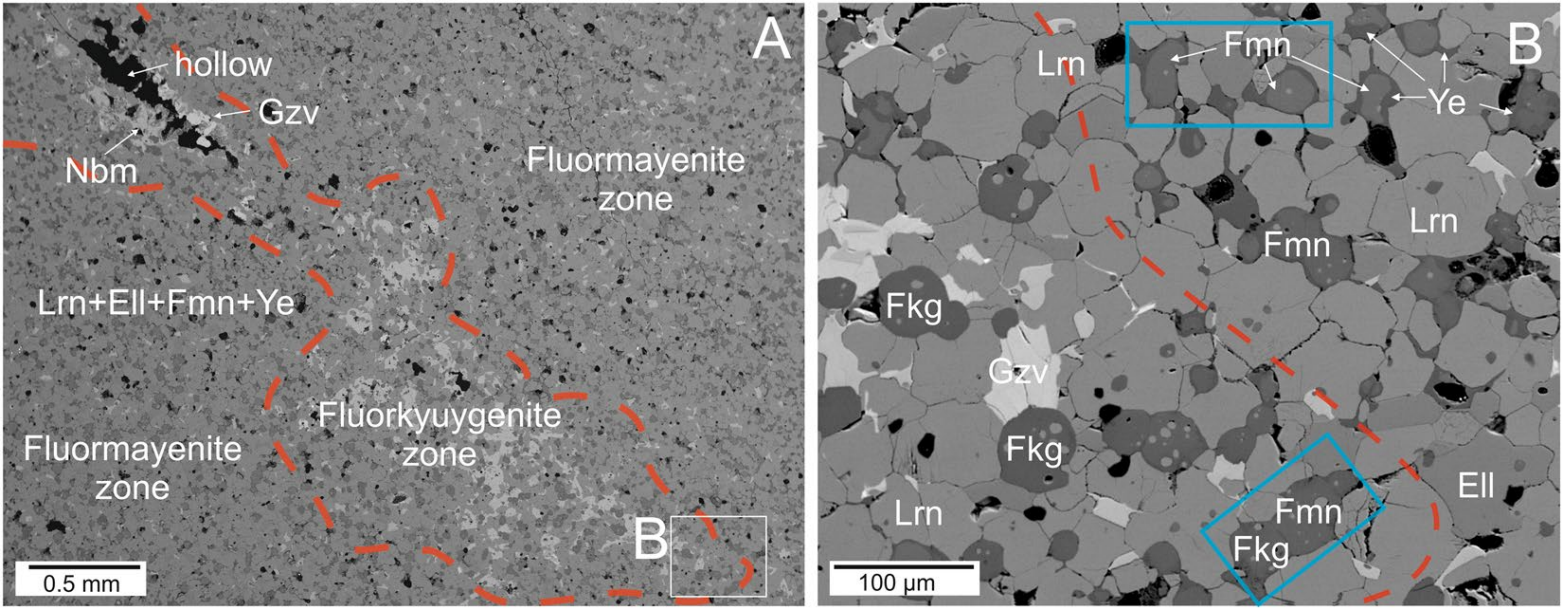
(**A**) Backscattered electron image of larnite rock with fluorkyuygenite in the porous linear zone and fluormayenite in the low-porous rock matrix. (**B**) Magnified fragment is shown in the frame in (**A**). The red line indicates the approximate boundary between fluorkyuygenite-bearing and fluormayenite-bearing zones. Grains, for which Raman mapping was performed, are shown in the blue rectangles. Ell – fluorellestadite, Fkg – fluorkyuygenite, Fmn – fluormayenite, Gzv -gazeevite, Lrn – larnite, Nmb – nabimusaite, Ye – ye’elimite.

Fluorkyuygenite associates with rare minerals such as nabimusaite KCa_12_(SiO_4_)_4_(SO_4_)_2_O_2_F, gazeevite BaCa_6_(SiO_4_)_2_(SO_4_)_2_O and ternesite Ca_5_(SiO_4_)_2_(SO_4_) (Fig. 2A,B). The composition of fluormayenite-bearing rocks is comparable to natural analogs of belite-sulfoaluminate cement clinkers because of similar composition and conditions of its formation^31^.

## Methods of Investigations

### Scanning electron microscopy (SEM) and Electron microprobe analysis (EMPA)

Scanning electron microscope Phenom XL (Faculty of Earth Sciences, University of Silesia) with EDS was used for initial studies of samples. Morphology and chemical composition of fluorkyuygenite, fluormayenite, as well as associated minerals, were studied. The chemical composition of mayenite group minerals was measured using electron microprobe analyzer CAMECA SX100/WDS (Institute of Geochemistry, Mineralogy, and Petrology, University of Warsaw). Electron probe microanalyses were performed at 15 kV, 20 nA and 1–2 μm beam diameter using the following lines and standards: Ca*Kα*, Si*Kα* – wollastonite; Al*Kα* – orthoclase, Cl*Kα* – tugtupite; Fe*Kα* – Fe_2_O_3_; Mn*Kα* – rhodochrosite; Ti*Kα* – rutile; Mg*Kα* – diopside; Na*Kα* – albite; F*Kα*, P*Kα* – apatite BB2; S*Kα* – barite, S*Kα –* barite.

### Confocal Raman spectroscopy (CRS)

Raman experiment was performed using WITec confocal Raman microscope CRM alpha 300M equipped with an air-cooled solid-state laser (λ = 532 nm) and an electron multiplying CCD (EMCCD) detector. The excitation laser radiation was coupled into a microscope through a single-mode optical fiber. To investigate the influence of the microscope configuration, a wide range of objectives (10×/0.2NA, 50×/0.5NA, 50×/0.76NA, 100×/0.9NA) at two different diameters of the pinhole (25 µm, 50 µm) were tested. Here, the 50×/0.76NA air Olympus MPLAN objective was chosen with the diameter of the pinhole equal to 25 µm as an optimal solution to preserve the compromise between lateral and depth resolution and ensure to avoid a loss in z-direction^32^. In this context lateral resolution (LR) estimated according to Rayleigh criterion LR = 0.61λ/NA, while depth resolution (DR) as DR = λ/(NA)^2^, where LR is the minimum distance between resolvable points (in X-, Y- direction), DR is the minimum distance between resolvable points (in the z-direction), NA is the numerical aperture, and λ is the wavelength of laser excitation. As a result, LR = 0.43 μm while DR = 0.92 μm. In turn, due to refraction effects and laser beam scattering observed in the deeper part of the sample, the DR values for minerals with n ≈ 1.6 determined according to equation proposed by Everall^33,34^ as follow: DR_−2μm_ = 1.13 μm, DR_−4μm_ = 2.26 μm, DR_−6μm_ = 3.39 μm, DR_−8μm_ = 4.53 μm (for more detailed information about lateral and depth resolution see Supplementary Material). Raman scattered light was focused onto a multi-mode fiber and monochromator with a 600 line/mm grating. Instrument calibration was verified by checking the position of the Si (520.7 cm^−1^).

Surface Raman imaging map was collected in a 150 μm × 100 μm area using 300 × 200 pixels (=60 000 spectra) with an integration time of 100 ms per spectrum, and precision of moving the sample during the measurements with a precision ±0.5 μm. These data were collected to distinguish different minerals of the mayenite group as well as co-associated phases. No special data about cracks system or grain size were taken into account. In turn, the stack scan option in Witec control software was applied to collect maps in-depth profiling up to 8 μm for every 2 μm in the z-direction from top to bottom of the sample whereas Raman signal was obtained from a volume of 100 × 100 × 8 μm^3^. Here, each map was collected in a 100 μm × 100 μm area using 200 × 200 pixels (=40 000 spectra) with an integration time of 100 ms per spectrum and moving the sample during the measurements with a precision ± 0.5 μm. The total exposure time for each map was estimated ca. 40 min.

All spectra were collected in the 200–4000 cm^−1^ range at 10 mW on the sample with 3 cm^−1^ spectral resolution. The output data were manipulated by performing a baseline correction using the auto-polynomial function of degree 3 and were submitted to an automatic cosmic rays removal procedure. Chemical images were generated by using a sum filter which integrating the intensity over a defined wavenumber range (hydroxyl region). This procedure was applied to preliminary recognize chemical and structural differentiation of the analyzed place and sample to further investigations. Then, the more detailed analysis was performed using cluster analysis (CA) to group objects (spectra from the map) into clusters. K-means analysis with the Manhattan distance for all Raman imaging maps was carried out to distinguish different minerals of the mayenite group from each other and co-associated minerals. The chemical analysis was performed for normalized spectra using WITec Project Plus Software. Finally, a band fitting analysis using a Lorentz-Gauss function with the minimum number of the component was done using the GRAMS 9.2 software package.

## Results

The chemical composition of the studied mayenite group minerals does not exhibit any essential differences regarding the content of main elements (Table 1, analyses 1–4). A difference of microprobe analysis totals is the only one observable variability. Grains located in the low-porosity zones are characterized by higher experimental totals (∼99%) than those from the high-porosity area (∼95%), measured at the same conditions (Table 1). Similar regularity was observed in individual zonal crystals, where total in the core is higher about 3–4% in comparison with the rim. Therefore, it is practically impossible to differ fluormayenite and fluorkyuygenite by chemical analyses on the base of EDS or WDS at different conditions that induced us to apply other technique. For comparison, the chemical composition of ye’elimite rims on fluormayenite grains is given in Table 1 (analysis 5).

**Table 1:** The chemical composition of fluorkyuygenite-fluormayenite mineral series (1–4) and ye’elimite rim (5) from Nahal Darga.

	1			2			3			4			5		
	Mean	s.d.	Range	Mean	s.d.	Range	Mean	s.d.	Range	Mean	s.d.	Range	Mean	s.d.	Range
	n = 8			n = 10			n = 3			n = 10			n = 10		
CaO	44.87	0.17	44.63–45.18	45.55	0.17	45.22–45.86	46.17	0.02	46.17–46.21	46.75	0.19	46.38–47.08	35.77	0.15	35.71–36.25
Na_2_O	0.25	0.03	0.22–0.29	0.26	0.02	0.24–0.29	0.25	0.03	0.24–0.31	0.31	0.02	0.27–0.34	n.d.		
SrO	n.d.			n.d.			n.d.			n.d.			0.54	0.04	0.48–0.61
Al_2_O_3_	47.11	0.17	46.81–47.39	47.70	0.23	47.36–48.09	48.28	0.09	48.22–48.40	48.92	0.24	48.56–49.38	48.65	0.28	47.83–48.75
Fe_2_O_3_	1.44	0.10	1.28–1.55	1.63	0.12	1.43–1.79	1.68	0.17	1.56–1.89	1.76	0.25	1.46–2.13	0.72	0.09	0.55–0.83
SiO_2_	0.05	0.06	0.20–0.01	0.07	0.4	0.02–0.13	0.12	0.03	0.11–0.16	0.15	0.03	0.09–0.21	n.d.		
SO_3_	n.d.			n.d.			n.d.			n.d.			12.48	0.12	12.24–12.66
F	1.59	0.09	1.47–1.77	1.74	0.09	1.56–1.80	1.68	0.11	1.54–1.74	1.62	0.12	1.40–1.76	n.d.		
Cl	0.21	0.01	0.18–0.22	0.21	0.01	0.18–0.22	0.20	< 0.01	0.19–0.20	0.18	0.01	0.16–0.20	n.d.		
H_2_O^*^	5.20			3.62			2.38			1.03					
-O = F + Cl	0.72			0.78			0.75			0.72			0.00		
Total	100.00			100.00			100.00			100.00			98.16		
calculated on 26 cations													calculated on 10 cations		
Ca	11.88			11.88			11.88			11.86			3.97		
Na	0.12			0.12			0.12			0.14			n.d.		
Sr	n.d.			n.d.			n.d.			n.d.			0.03		
X	**12**.**00**			**12**.**00**			**12**.**00**			**12**.**00**			**4**.**00**		
Al	13.72			13.68			13.67			13.65			5.94		
Fe^3+^	0.27			0.30			0.30			0.31			0.06		
Si	0.01			0.02			0.03			0.04			n.d.		
Z	**14**.**00**			**14**.**00**			**14**.**00**			**14**.**00**			**6**.**00**		
S	n.d.			n.d.			n.d.			n.d.			0.97		
F	1.24			1.34			1.28			1.21			n.d.		
Cl	0.09			0.09			0.08			0.07			n.d.		
OH^*^	8.57			5.88			3.81			1.63					

^*^Water was added to 100% weight of the measurement; n.d. - not detected, s.d. - standard deviation.

Thus, to supply the chemical analyses and try to understand problems connected with EDS or WDS investigations, Raman spectroscopy has been applied. It should be noted that calcium-aluminates are a subjects of intense Raman spectroscopy investigations for long time^35–37^, especially due to the importance of CaO-Al_2_O_3_ system in geochemical and material sciences^38^. The investigations based on a single measurements did not allow to obtain reliable depiction of the analyzed probe. A lot of analyses had to be performed to get a full data set on the whole sample. To meet the scientific expectations and to solve a problem of time-consuming measurements, Raman imagining (2D, 3D) was developed to investigate the chemical distinctions in more details. This approach was used to elaborate a model of a different route of water incorporation into mineral crystal structure or follow the spatial differentiation of various minerals. Finally, combining chemical analyses and spectroscopic data allows to understand and resolve a problem formation of minerals of the mayenite group as well as to describe a genesis of host rocks.

### 2D Raman imaging

**Figure 3:**
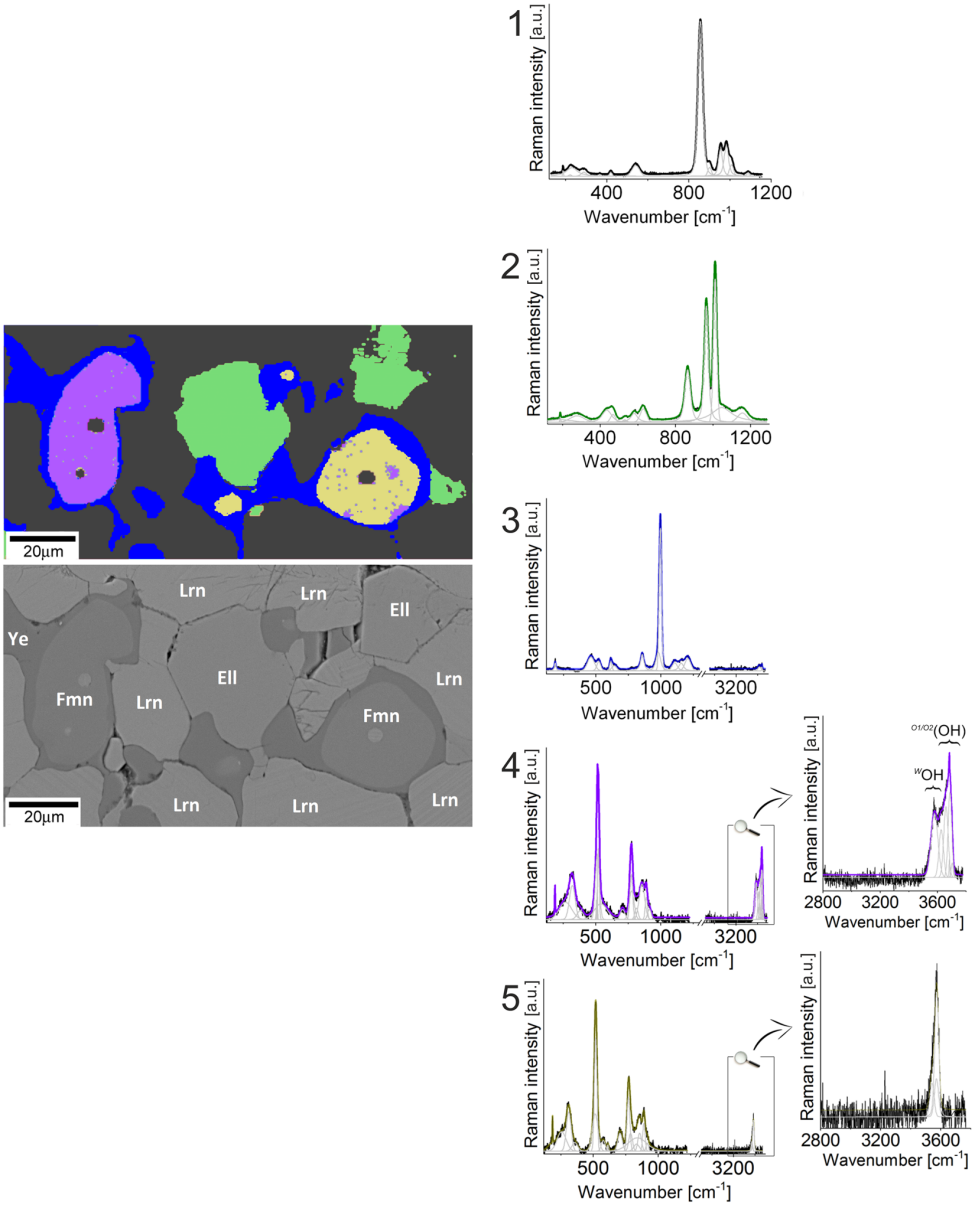
*K*-means analysis performed in a fragment of the sample from the border of low-and high porous zone. Cluster tree with the root and the first level clusters determine five different areas. The black cluster (1) relates to larnite matrix, the green- (2), blue- (3), violet- (4) and yellow (5) clusters correspond to fluorellestadite, ye’elimite, defected fluormayenite, fluormayenite, respectively. Raman spectra of larnite and ellestadite were visualized in the 200–1250 cm^−1^ range while other spectra in the full range from 200–3750 cm^−1^ with a focus on hydroxyl range from 2800–3750 cm^−1^. The cluster combines Raman image was correlated with SEM picture. Ell - fluorellestadite, Fmn - fluormayenite, Lrn - larnite, Ye - ye’elimite.

*K*-means cluster analysis due to 2D Raman imaging allowed to select five average spectra of different minerals. Three of them are associated with larnite, fluorellestadite, and ye’elimite, whereas the other ones are assigned to fluormayenites (Fig. 3). To shed more light on the structural features of these minerals, each spectrum was fitted using the band fitting procedure with application of a minimum number of components. This approach is extremely important for data interpretation as that enables to find modes associated with vibrations of main functional groups built into the crystal structure of identified minerals. However, the spectra of larnite, fluorellestadite, and ye’elimite are less important and therefore do not considered in detail, while a spectral analysis performed on fluormayenites was carried out with more diligence.

**Figure 4:**
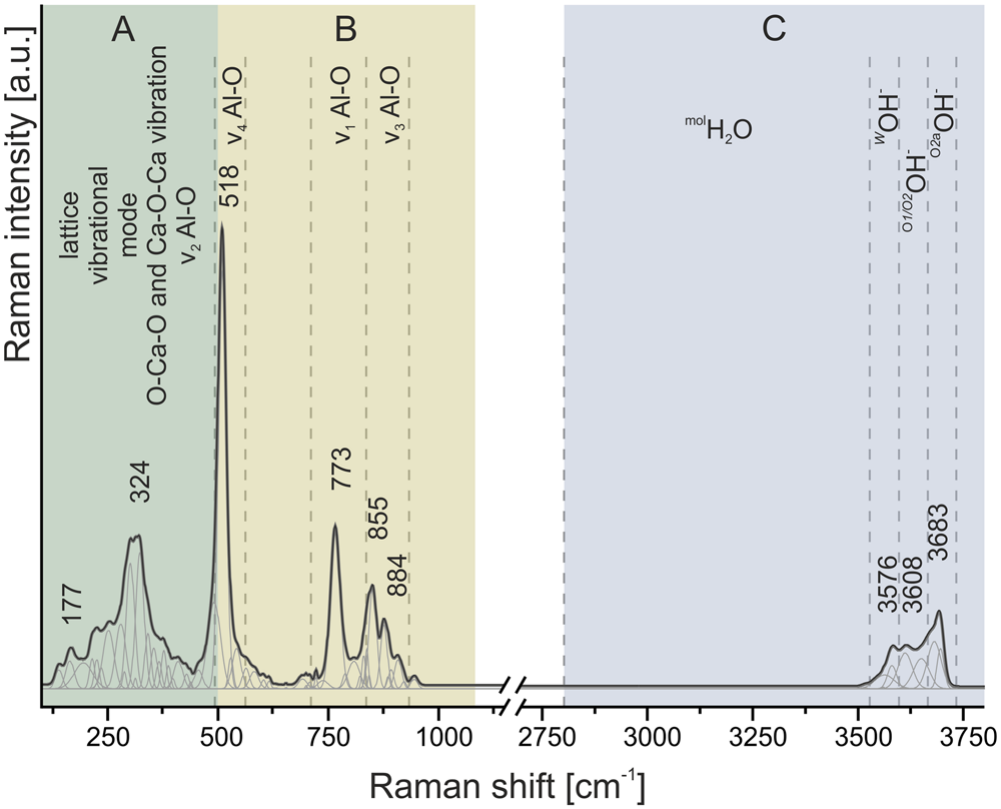
An example of fluormayenite spectrum with marked ranges corresponding to particular types of vibrations.

First of all, it is crucial to identify the origin of bands observed in the Raman spectrum of fluormayenite. The typical Raman spectrum of fluormayenite was fitted using Gauss function with preservation of a minimum number of component and divided into three spectral ranges: (A) 150–500 cm^−1^, (B) 500–1100 cm^−1^ and (C) 2800–3750 cm^−1^ (Fig. 4). The low-wavenumber range (A) indicates a group of low-intensity bands associated with the lattice mode – deformational ones of calcium in octahedra^39^ or vibrations within Ca[AlO_4_] units^3^. The range (B) includes vibrations of the fluormayenite structure backbone such as stretching and bending vibrations of Al-O in (AlO_4_)^5−^ tetrahedra. Here, the bands were ascribed to three ranges: 506–518 cm^−1^, 706–776 cm^−1,^ and 855–890 cm^−1^, related to ν_4_, ν_1_ and ν_3_ modes of Al-O in [T_1_O_4_] and [T_2_O_4_], respectively^7^. The ν_2_ vibration of Al-O are located near 310 cm^−1^, but due to overlapping with other types of vibration, the accurate band assignment can be affected by error. Additional bands in every spectral region (Fig. 4) come from the different symmetry of tetrahedral units depending on aluminium occupancy in T_1_ or T_2_ sites^40^. According to previous reports, the fluormayenite-type structure belongs to deformational-sensitive one because of comparatively easy elemental substitution Fe^3+^, Si → Al, OH^−^ → Cl^−^, F^−^ etc. Therefore, additional low-intense bands are observed not only in fluormayenite structure but in general for minerals of the mayenite group reported in the literature^5,7,21^. These low-intense bands can be an evidence of structural deformation causing by water incorporation^41^. and formation of Ca-OH^42^ or Al-OH…O-Al bonds^43^. Finally, a range (C) associated with different type of water incorporated into the crystal structure seem to be the most indicative and it is diagnostic for identification and differentiation of fluormayenite and hydroxylated fluorkyuygenite. The character of spectra in 3500–3750 cm^−1^ region is variable and intensity and position of particular bands located in this interval differ in particular crystals. The bands in ^mol^H_2_O region are observed in fluorkyuygenite spectra (Fig. 4C). The position and full width at half maximum (FWHM) of all minerals of the mayenite group recognized during the experiment summarized in Table S1 (Supplementary Data). Consequently, mayenite can be described by three different groups of modes related to various OH vibrations. The first group of bands consists of the components with broad line widths located at 2800–3450 cm^−1^ corresponding to symmetric and anti-symmetric stretching vibrations of molecular water and is characteristic for kyuygenite-type minerals (bands not present on fluormayenite spectrum, see Fig. 4)^5,7^. The next group of bands located in the 3550–3600 cm^−1^ range with a maximum at ca. 3573 cm^−1^ is connected with a presence of stretching vibrations of hydroxyl groups occupying the *W* site (typical for mayenites, Fig. 4)^44,45^. In turns, the nature of intense bands above 3600 cm^−1^ is still not properly explained. One of the hypotheses suggests that there is an evidence of surface absorbed water or remnant of hydrogarnet defects as a part of the incomplete transformation of minerals of the mayenite group into hydrogarnet^21^. The third group with the band at 3680 cm^−1^ is related to substitution of O^2−^ by OH groups in tetrahedra according to the scheme: O^2−^ + F^−^/Cl^−^ → 3(OH)^−^ resulting in a change of aluminium coordination from tetrahedral to octahedral^3,5,46^. Hence, mineral with bands in that region can be considered as a hydrated “defected” phase, where hydroxyl groups are located in various structural configurations (observable in some group of mayenites, Fig. 4). This phase, probably, belongs to the structure which contains more water than typical fluormayenite (compare violet and yellow spectra in Fig. 3). Such assignment was also performed according to the data presented before in literature for the similar phases^5,7^.

According to these data and based on the *K*-means cluster analysis of non-porous zone, within the analysed grains one can distinguish two Raman spectra with bands arrangement in the fingerprint region characteristic for minerals of the mayenite group and without any special differences in band position or FWHM. However, more detailed investigation of hydroxyl region showed some discrepancies in a number of bands suggesting a presence of two different minerals. The band arrangement for the first phase is ascribed to structurally defected and strongly hydrated fluormayenite (violet in Fig. 3), while the number of bands and its position of the second phase is typical for fluormayenite with OH^−^ → F^−^ (yellow in Fig. 3). These results correspond well to the differences in totals obtained from a microprobe analyses (Table 1, analyses 3 and 4). Moreover, according to Raman imaging data, more hydrated fluormayenite presence correlates with the discontinuities in ye’elimite rims. In turn, this information might be correlated with an effect of significant incorporation of water into the crystal structure of fluormayenite.

### 3D Raman imaging

**Figure 5:**
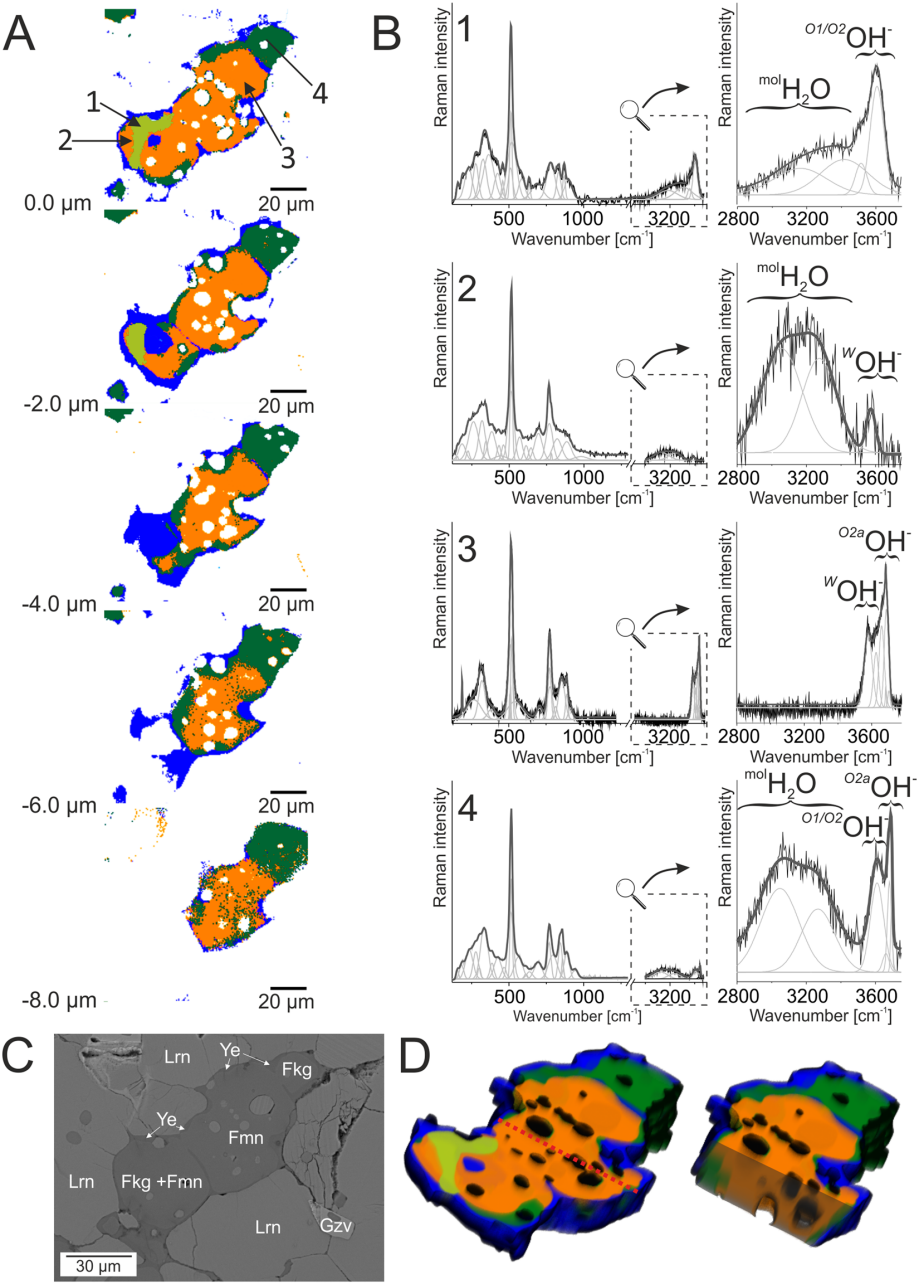
(**A**) K-means cluster analysis with the root clusters collected in the depth profile stack scan range from 0 to −8 μm and different cluster colors correspond to different phases: dark green and orange clusters come from fluorkyuygenite and fluormayenite, respectively, light green cluster means hydrated fluorkyuygenite, blue cluster determines ye’elemite and white cluster means larnite matrix. (**B**) Raman spectra of minerals of the mayenite group visualized in the 200–3750 cm^−1^ range with magnified the hydroxyl range from 2800–3750 cm^−1^. Spectrum 1 - hydrated fluorkyuygenite from the center of the hydrated zone, spectrum 2 - fluorkyuygenite from the rim of the hydrated zone, spectrum 3 - fluormayenite, spectrum 4 - typical fluorkyuygenite. (**C**) BSE image of the mapped area. Fkg - fluorkyuygenite, Fmn - fluormayenite, Gzv - gazeevite, Lrn - larnite, Ye - ye’elimite. (**D**) 3D model of examined grains composed of cluster analysis. The whole grain is presented on the left side, on the right side intersection, indicated by red line, is shown.

3D Raman imaging with stack scan mode was carried out to illustrate a way of water incorporation into different types of fluorine-bearing minerals of the mayenite group depending on ye’elimite rim thicknesses, as well as spatial distribution of minerals (Fig. 5A). The analysis was carried out on the fluormayenite-fluorkyuygenite grain located at the contact of low- and high-porosity zones (Fig. 1). In addition, the size of cracks and channel system, the habit of crystals, its size, structural and chemical homogeneity of fluormayenites and fluorkyuygenites were investigated using confocal mode. It is important to note, that this information cannot be obtained in the case of typical 2D Raman imaging. In this context, five Raman images on specimens from the porous zone were collected beginning from the surface up to −8 µm. The post-processing *K*-means cluster analysis revealed a presence of larnite, or other co-associated phases as well as five different Raman spectra of fluormayenite-type minerals. However, the most interesting conclusions were provided by the analyses of minerals of mayenite group derivatives. Hence, a detail observation done for fluorine mayenites pointed out to small differences in bands intensities in the region of 855–890 cm^−1^, which may correlate with alteration in the number of hydroxyl groups occupying the *W* site. It may be associated with the greater impact on Al-OH…O-Al unit^43^ in the crystal structure of hydrated phases in relation to the typical mayenite (compare band position and FWHM in Table S2). Another bands observed in the fingerprint region are practically not structurally affected by the presence of water (not visible changes in band position and FWHM, Fig. 5, Table S2). In turn, the most noticeable changes found within the hydroxyl region pointed to the various type of water occurred in the crystal structure of fluorine derivative of mayenite and strictly depends on the thickness or discontinuities of ye’elimite rim, cracks, or channel systems (Fig. 5). Hence, similar to the 2D imaging analysis, one can conclude that the presence of discontinues rim might provide easier incorporation of water into the crystal structure of minerals of the mayenite group and formation of strongly hydrated water clathrate phase - fluorkyuygenite. In turns, the ye’elimite rim becomes thicker and more continues, the impact of molecular water incorporated into the fluormayenite-type structure is lower. In consequences, one can observe the preserved origin phase in an unhydrated or low hydrated form containing OH-groups only in the *W* site (Fig. 5).

The position of bands in the hydroxyl region for all minerals separated due to *K*-means cluster analysis are summarized in Table S2. Here, Raman spectrum of fluormayenite is characteristic for other minerals of the mayenite group with hydroxyl band centred at 3571 cm^−1^. It is interesting, that such spectrum is affected by the presence of low intense bands between 2800–3400 cm^−1^ resulted from molecular water occupying the *W* site, probably due to the crystallization of such phase in the vicinity of micro-cracks or channels which constitute the flow path of water through the rock system (spectrum 1 in Fig. 5). According to the *K*-means cluster analysis, it was found that water content in fluormayenite structure significantly increases in the marginal part of grain, indicating a greater role of cracks or small discontinues in ye’elimite rim. As a result, typical bands of the hydroxyl vibration region, as well as stronger bands in its intensity corresponding to vibrations of molecular water, appear in the Raman spectrum (spectrum 2 in Fig. 5). Furthermore, the greater share of molecular water into the structure indicates structural distortion in the cages which are illustrated by the OH-band position at 3574 cm^−1^. In both phases, there are no bands related to hydrogarnet defects.

Additionally, according to 3D Raman imaging and *K*-means cluster analysis, it was determined that investigated minerals are spatially limited and form a lenticular body, restricted by ye’elimite (see blue area in Fig. 5A). As a result, the process of water incorporation into the mayenite structure can be presented in a different way. In marginal part of fluormayenite crystals, close to ye’elimite rim, a greater impact of water incorporated into the mineral structure is observed. For instance, molecular water, and hydroxyl groups in different structural configurations appeared when ye’elimite rim around fluormayenite grain is fine or absence. It is confirmed by the presence of bands at the 2800–3450 cm^−1^, 3610 cm^−1^ and 3680 cm^−1^. Moreover, bands arrangement characteristic for hydroxyl and molecular water resulted from a presence of cracks and channels are related to a complete hydration. That process provides formation of typical fluorkyuygenite characterized by the presence of OH-groups, molecular water, and hydrogarnet defects (see spectrum 4 in Fig. 5). In turn, the main phase distinguished by *K*-means cluster analysis located in the central part of the grain indicates a presence of fluormayenite with predominance of bands correlated to vibrations of typical hydroxyl group in the *W* site and vibrations of OH^−^ within the hydrogarnet or mayenite defects. Hence, the band arrangement is typical for the spectrum of defected fluormayenite and might be comparable with similar one reported for chlormayenite^21^. Moreover, three hydrated phases present the main part of analysed grain without spatial diversity (see orange and green clusters in Fig. 5). A similar observation was noted in the case of thick ye’elimite rim occurred around the analyzed grains. It confirmed the occurrence of all fluorine derivatives of minerals of the mayenite group in the whole volume of the host rock.

**Figure 6:**
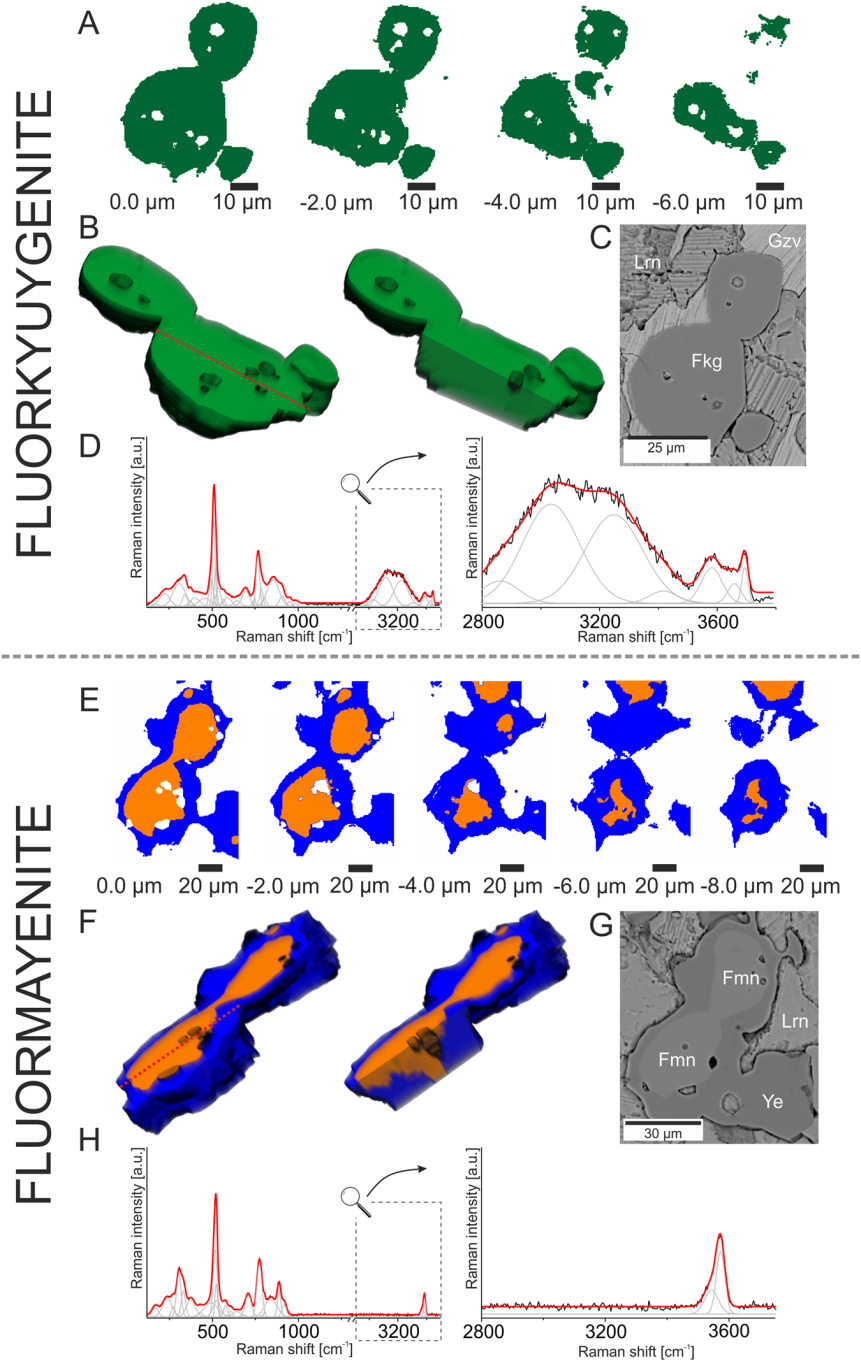
(**A**) *K*-means cluster analysis collected in the depth profile stack scan range from 0 to −8 μm of fluorkyuygenite (green cluster). (**B**) 3D model of fluorkyuygenite composed of cluster analysis. (**C**) BSE image of mapped fluorkyuygenite. (**D**) Raman spectrum of fluorkyuygenite visualized in the 200–3750 cm^−1^ range with the magnified hydroxyl range 2800–3750 cm^−1^. (**E**) *K*-means cluster analysis collected in the depth profile stack scan range from 0 to −8 μm of fluormayenite. Fluormayenite is marked as orange cluster, blue cluster correspond to the ye’elimite. (**F**) 3D model of fluormayenite composed of cluster analysis. (**G**) BSE image of mapped fluormayenite. (**H**) Raman spectrum of fluormayenite visualized in the 200–3750 cm^−1^ range with the magnified hydroxyl range 2800–3750 cm^−1^. Fkg - fluorkyuygenite, Fmn - fluormayenite, Gzv - gazeevite, Lrn - larnite.

Finally, to show differences between minerals of the mayenite group from the medium- and low-porosity zones, 3D imaging was also performed for fluorkyuygenite and fluormayenite from the zone center (Fig. 6). In case of such analysis the zonation is absent, while fluorkyuygenite and fluormayenite are characterized by the constant distribution of water incorporation. As a result, only one spectra cluster in fluormayenite or fluorkyuygenite is generated.

## Discussion

The cluster analysis performed on the basis of 2D Raman imaging allowed to distinguish fluormayenite grains with a different type of OH-bands observed at the 3450–3750 cm^−1^ range, wherein 3D Raman imaging allowed to identify not only fluormayenite, but also fluorkyuygenite with bands related to H_2_O at 2800–3400 cm^−1^. As a result of a combination of Raman spectroscopy with micro-chemical analyses, differences of water distribution in minerals with the fluormayenite-type structure from porous zones can be estimated. Here, four types of water incorporation into minerals of the mayenite group could be distinguished. The first type of water incorporation is connected to fluorkyuygenite characterized by weight loss of ∼5 wt. % (Table 1, analysis 1) and Raman bands at 2800–3450 cm^−1^, related to neutral water molecules in the structural cages and two low-intense peaks at 3570 and 3680 cm^−1^ corresponding to OH^−^ substitution. The second type of water incorporation is related to fluormayenite (Table 1, analysis 2) with ∼3.5% weight loss, which at the higher weight loss (c.a. 4%) still has “kyuygenite-type” water, but its content is too low to classify this mineral as fluorkyuygenite. In this phase, the band at 3680 cm^−1^ is still present, 3570 cm^−1^ band show drop in intensity, and the new band at 3610 cm^−1^ appears. The third type of water incorporation corresponds to fluormayenite (∼2.3% weight loss; Table 1, analysis 3) with three distinct bands at 3570, 3610 and 3680 cm^−1^. The fourth type of water incorporation is connected to fluormayenite with the smallest weight loss (≤ 1 wt. %; Table 1, analysis 4) with one band at 3570 cm^−1^.

To determine a water type in the fluormayenite structure on the basis of Raman spectroscopy, the calculation of theoretical content of the defined water types for microprobe analyses is possible only when one or two types of water are present in the analysed structure. The calculation is impossible in the case of more complicated configurations.

**Figure 7:**
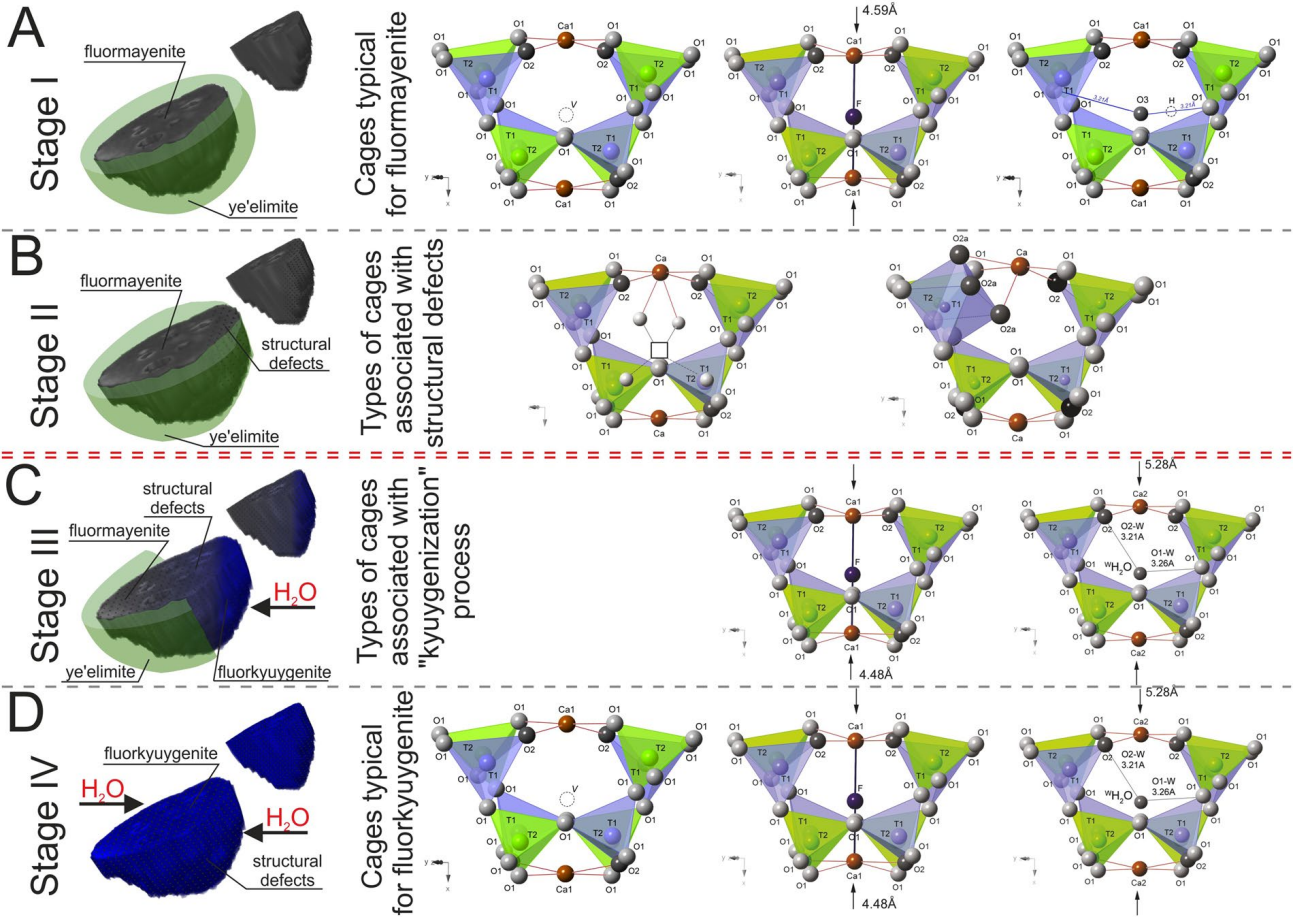
Four stages of water incorporation into fluormayenite structure. The double red line separates two thermal events.

3D Raman imaging with correlation to diffraction data give an opportunity to create a theoretical model of water incorporation into the minerals of mayenite group structure. It should be noted that structural considerations were based on the published data on fluormayenite and fluorkyugenite^5^. The processes caused by structural modification can be correlated with water presence in the environment as well as the thermal history of the pyrometamorphic complex which may divide into four main stages (Fig. 7).

The first stage (Fig. 7A) was connected to the formation of fluormayenite with typical atom arrangement, in which three types of cage occupation can be distinguished: (1) fluorine in the *W* site, (2) hydroxyl group in the *W* site and/or (3) unoccupied the second one with the vacant *W* site. The size of these cages showed that Ca-Ca distance in fluormayenite is about 4.59 Å (1), 5.00 Å (2) and 5.64 Å (3)^5^. The authors also suggested that water may incorporate into the structure of fluormayenite as a result of isomorphic substitution according to the scheme ^*W*^F^−^ → ^*W*^OH^−^ or, alternatively, ^*W*^▯ → ^*W*^OH^−^ explaining cage size about 5.00 Å. Moreover, formation of such mineral may correspond to high temperature process, usually taken place at temperatures above 900 °C. This statement finds confirmation in literature, describing a replacement of the small amount of fluorine by hydroxyl^5^. It is worth to note that very small area (few μm) of studied minerals excludes the possibility of X-ray measurements and only one technique predispose to structural investigation is Raman spectroscopy. As a result of such approach, fluormayenite in the first stage of formation seems to be deprived of the opportunity to form clearly marked H-bonds because of the negligible amount of water present in the system. This suggestion is supported by the presence of thick ye’elemite rim which is practically not affected through some discontinuities or cracks. Such phase can be considered as a most primary fluormayenite with low hydration degree (yellow spectrum in Fig. 5). In turn, the presence of fluormayenite-type minerals with low intense bands at ~3200, ~3400, ~3450 and ~3550 cm^−1^, related to water in different structural configuration is an effect of discontinue ye’elemite rim and correlates with a development of breaks or cracks in the massive rock system (spectra 1, 2 in Fig. 5). That leads to the formation of hydrated fluormayenite with atypical structural atom arrangement (I/II stage in Fig. 7A,B). What is more, hydroxyl ions can migrate inside the crystal structure and modify atom arrangement in a different way. The one of possible route is associated with the structural transformation of the aluminum coordination from tetrahedral to octahedral, according to the scheme ^*W*^F^−^ + 2 ^*W*^▯ + ^*2O*^O^2−^ → ^*2Oa*^3(OH)^−^ + 3 ^*W*^▯^3,5^, provide increasing the Ca-Ca distance up to 5.70 Å^5^. It gives at the same time an opportunity to hydrogen bond formation due to the donor-acceptor distance between hydrogen and one of the oxygen from the aluminum polyhedra (Fig. 7B). The presence of ^*W*^OH presume an existence of two O-O distances: *O*1 − *W* = 3.26 Å, *O*2 − *W* = 3.29 Å, while appearance of ^*O2a*^ОН groups points out two O–O distances: *O*2a − *O*1 = 2.92 Å, *O*2a − *O*1 = 2.82 Å^5,7^, which according to Libowitzky may correspond to medium strong hydrogen bonds^47^. In the Raman spectrum one can find that some bands in the hydroxyl stretching region can be linked to electrostatic interaction with attracting force between proton-donor and oxygen-acceptor. It is also worth to note that low intense and broad Raman bands observed in the 3200–3400 cm^−1^ region can be probably associated with the surface water absorption as an effect of a strongly microporous nature of fluormayenite-type phase. Stage II as a path of faster migration of water, imply the formation of fluormayenite-type phases with hydrogarnet defects (Fig. 7B), described previously in chlormayenite^21^ and Sr-hydrogarnet^15^. It is correlated with Raman imaging indicating that the main part of the image is associated with this phase (orange cluster in Fig. 5). Moreover, the appearance of hydrogarnet defects is probably linked to lowering temperature prevailing in the massive complex and might be revealed as a close to 250–300 °C^15,21^. Stage III, in turn as a route of relatively fast water migration due to very thin ye’elemite rim, results in significant increase of band intensity of the 3200–3400 cm^−1^ range, assigned to the vibrational modes of molecular water (Fig. 7C). This observation is connected to a relatively strong hydration of a system according to the scheme ^*W*^▯ → H_2_O. However, previously described diffraction data suggest that molecular water partially occupy the same position as fluorine^5^. As a result, this process assumes a decreasing of the original cage size (5.64 Å empty)^5^ as a result of the calcium atom tending towards to the center of the cage. In consequence, the dimension of the cage estimated from the single crystal data of fluorkyuygenite and XRD refinement with fluorine in *W* site is equal to 4.48 Å, while ^*W*^H_2_O is 5.28 Å^5^ (Fig. 7C). It may imply formation of H-bond as О···Н-^*W*^O-Н···О (*W* = cage center). However, it is not clear to predict the real configuration of the hydrogen bond because of a large number of oxygen sites (16) which define the inner surface of the structural cage. Hence, according to single crystal data reported in literature^5^, one can find three main possible configurations of O-O distances: (1) *O*1 − ^*W*^O = 3.21 Å with *O*1- ^*W*^O -*O*1 angle ≈ 150.9°, (2) *O*2 − ^*W*^O = 3.26 Å with *O*2- ^*W*^O -*O*2 angle ≈ 118.4°, and (3) *O*2 − ^*W*^O = 3.26 Å, *O*1 − ^*W*^O = 3.21 Å with *O*2- ^*W*^O -*O*1 angle ≈ 103.9° which are the closest one to the H-O-H angle configuration in dipole H_2_O molecule. According to Libowitzky such proton-acceptor bond distance may activate symmetric and anti-symmetric stretching vibration of molecular water observable in the 2800–3600 cm^−1^ region^47^. Furthermore, such different possibilities of H-O-H bond distances may translate into a broadening of bands at this region. What is more, a significant increase in the water in the environment correlates to disappearance of ye’elemite rim and provide appearance of structural defects in the crystal structure of fluormayenite. It is also associated with gradually increase the intensity of water and hydrogarnet bands in relation to the typical fluormayenite ones (spectra 3, 4 in Fig. 5). Such stage is also responsible for “kyuygenitization” process and the transformation of low-water content fluormayenite into high-water content fluorkyuygenite. According to literature data^5^, this process as a result of gaseous metasomatism occurs at lower temperature, usually below 400 °C and is an indicator of gradually lowering temperature of the whole rock complex. Such phases are usually connected to the marginal part of grain, probably due to not fully structural transformation of fluormayenite-type phase. It might also be linked to gradually alteration of pyrometamorphic larnite rocks by water vapor enriched gases which will lead with time to transformation of fluormayenite to fluorkuyugenite with presence of all mentioned above structural water arrangements inside the cages (IV stage in Fig. 7D). As a result, these transformation conditions might be related to the reconstruction the thermal history of the whole rock complex.

Finally, from a mineralogical point of view, a presence of gazeevite and nabimusaite in the fluorkyuygenite-bearing zones can prove a thermal events in the past. Both minerals form as a products of high-temperature alternation involving melts or gases connected with combustion process^30,48^. Taking into account, that distribution of gazeevite and nabimusaite is restricted to the same porous zones, which include fluorkyuygenite, it may be assumed, that porous zone was thermal altered.

## Conclusions

Raman imaging was for the first time applied to distinguish variability within the mayenite group minerals, that it was not possible using typical methods as electron microbeam apparatus with EDS or WDS. It was shown that presence or absence ye’elimite rim is responsible for crystallization of H_2_O-free fluormayenites or fluorkyuygenites, respectively. As a result, these data gave an opportunity to elaborate a model of water incorporation into the fluormayenite structure. It is assumed that fluormayenite formed as a primary phase. During retrogression it was replaced by fluorkyuygenite as a result of influence of fluids or gases enriched in H_2_O. It has been shown that certain types of water and OH^−^ substitutions arise from more than one thermal event. Hence, the first event was associated with the formation of typical fluormayenite at the temperature above 900 °C while the defects presence in the structure was correlated with cooling of the rock system (Stage I–II in Fig. 7A,B). In turn, the second low-temperature event with high water availability caused the formation of fully hydrated fluorkyuygenite (Stage III–IV in Fig. 7C,D). Finally, 3D Raman imaging was used to observe a spatial diversity of fluormayenite-like phases as well as to determine the size of channel systems and zonation ascribed to the individual phases, which occurred in the vicinity of the porous system.

## Electronic supplementary material

Supplementary Information
