# Supplementary material for: Raman imaging as a new approach to identification of the mayenite group minerals
Source: Sci Rep. 2018 Sep 11;8:13593. doi: 10.1038/s41598-018-31809-4 (PMC6134120; doi:10.1038/s41598-018-31809-4)
Supplement: Supplementary file 1 — Supplementary Information [file 41598_2018_31809_MOESM1_ESM.doc]

# Raman imaging as a new approach to identification of the mayenite group minerals

Środek D.^1^, Dulski M.^2,3^, Galuskina I.^1^

*^1^ Faculty of Earth Sciences, Department of Geochemistry, Mineralogy and Petrography, University of Silesia, Bedzińska 60, 41 - 200 Sosnowiec, Poland*

*^2^ Institute of Material Science, University of Silesia, Uniwersytecka 4, 40-007 Katowice, Poland*

*^3^ Silesian Center for Education and Interdisciplinary Research, 75 Pułku Piechoty 1a, 41 - 500 Chorzow, Poland*

*Correspondence to dsrodek@us.edu.pl*

**Supplementary information**

**Lateral and depth resolution discussion**

The crucial features of confocal Raman system, which have to be taken into account during the experiment, is the appearance of multi-optical effects as a result of the laser-solid interaction (i.e. reflection, refraction index, etc.). The sample data interpretation turned out to extremely important through the depth profiling studies and have to be discussed more in detail.

Hence, one can address that the spatial resolution depends on the 3 main factors: (1) the laser wavelength (λ), (2) the objective numerical aperture (NA), which determine the size of the laser focus volume, and (3) the diameter of the pinhole (d_0_) through which the scattered light is guided back into the spectrometer and determines the degree of confocality. In this context, the lateral resolution (LR) may be estimated according to Rayleigh criterion LR = 0.61λ/NA. In turn, laser wavelength and NA are determined by the size of the laser focal volume and the volume from which the spectrum is recorded. Here, higher laser wavelengths usually provide an increase a focal volume and the same decrease the resolution. Moreover, the resolution increases with increasing NA as well as with decreasing diameter of the pinhole. As a result, estimation of the depth resolution is more complicated, especially when the laser beam is become focused more deeply into a sample as in the case of a mineralogical sample which is usually transparent, semi-transparent or opaque and features of different refractive-index (n) values. Here, the application of the objectives with high NA cause that the focal plane is usually localized more deeply into the sample than the displacement from the objective yielding a worse depth resolution. Depth resolution is also linked to the difference between the maximum and minimum depths of focus (∆) and is correlated with scattering effect of the laser beam which makes the focus more diffuse. It provides degradation of the depth resolution, especially in the deeper part of the sample. Hence, in the case of mineralogical samples, important is to correlate wavelength, and NA value, with a suitable diameter of the pinhole as well as physical parameters of the sample to ensure as high depth resolution in the real experiment as it can be done and minimize the effect of laser diffusion. However, still, it is practically not possible to avoid the broadening of the focal volume with increasing depth and the rejecting light from out-of-focus regions which ensure decreasing the signal intensity with increasing depth. Hence, the change of the spectral intensity with penetration of the laser beam into the sample has to be considered each time during the data analysis of inorganic materials. Concluding all of that problems, the real depth resolution during the studies of mineralogical objects each time ought to be calculated using equation proposed by Everall^1,2^:


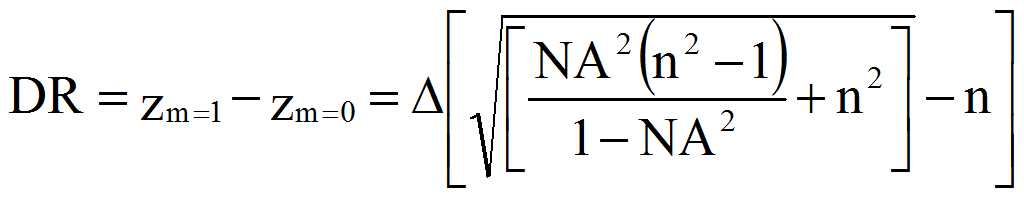


Lateral resolution (LR) was estimated according to Rayleigh criterion LR = 0.61λ/NA, where LR is the minimum distance between resolvable points (in X-, Y- direction), NA is the numerical aperture, and λ is the wavelength of laser excitation. In turns, depth resolution (DR) of an optical microscope is proportional to DR = λ/(NA)^2^, where DR is the minimum distance between resolvable points (in z-direction). As a result, LR = 0.43 μm while DR = 0.92 μm for excitation laser line (λ = 532 nm) and air objective (50x /0.76NA). In turn, due to refraction effects and laser beam scattering observed in the deeper part of the sample, the DR values for minerals with n ≈ 1.6 determined as follow: DR_-2μm_ = 1.13 μm, DR_-4μm_ = 2.26 μm, DR_-6μm_ = 3.39 μm, DR_-8μm_ = 4.53 μm

1. Everall, N. J. Confocal Raman Microscopy: Why the Depth Resolution and Spatial Accuracy Can Be Much Worse than You Think. *Appl. Spectrosc.* **54,** 1515–1520 (2000).

2. Everall, N. J. Modeling and Measuring the Effect of Refraction on the Depth Resolution of Confocal Raman Microscopy. *Appl. Spectrosc.* **54,** 773–782 (2000).

Table S1 Band position and corresponding FWHM obtained on the basis of peak fitting analysis for spectra of cluster analysis on the basis of surface Raman imaging (see Fig. 2). Peak fitting was done using Voigt function uploaded into Grams software package. Hydroxyl region was bold, bands of stretching vibration of water were additionally italic.

| Spectrum 1 | | Spectrum 2 | |
| --- | --- | --- | --- |
| Band position [cm^-1^] | FWHM | Band position [cm^-1^] | FWHM |
| *-* | *-* | 176 | 62 |
| 187 | 5 | 187 | 5 |
| 268 | 112 | 258 | 82 |
| 321 | 52 | 312 | 43 |
| *-* | *-* | 359 | 52 |
| 381 | 58 | *-* | *-* |
| 513 | 36 | 515 | 52 |
| 520 | 15 | 519 | 23 |
| 540 | 116 | *-* | *-* |
| *-* | *-* | 578 | 36 |
| *-* | *-* | 612 | 12 |
| 707 | 42 | 707 | 40 |
| 774 | 25 | 771 | 25 |
| 777 | 44 | 788 | 34 |
| 814 | 30 | 834 | 51 |
| *-* | *-* | 840 | 167 |
| 856 | 50 | 858 | 33 |
| 889 | 20 | 890 | 22 |
| 913 | 31 | 915 | 21 |
| *-* | *-* | ***3541*** | ***32*** |
| ***3579*** | ***55*** | ***3573*** | ***29*** |
| ***3626*** | ***37*** | *-* | *-* |
| ***3659*** | ***36*** | *-* | *-* |
| ***3683*** | ***4*** | *-* | *-* |
| ***3687*** | ***29*** | *-* | *-* |
| ***3702*** | ***13*** | *-* | *-* |

Table S2 Band position and corresponding FWHM obtained on the basis of peak fitting analysis for spectra of cluster analysis on the basis of surface Raman imaging (see Fig. 3). Peak fitting was done using Voigt function uploaded into Grams software package. Hydroxyl region was bold, bands of stretching vibration of water were additionally italic.

| Spectrum 1 | | Spectrum 2 | | Spectrum 3 | | Spectrum 4 | | Spectrum 5 | |
| --- | --- | --- | --- | --- | --- | --- | --- | --- | --- |
| Band position [cm^-1^] | FWHM | Band position [cm^-1^] | FWHM | Band position [cm^-1^] | FWHM | Band position [cm^-1^] | FWHM | Band position [cm^-1^] | FWHM |
| 156 | 28 | 153 | 11 | 156 | 28 | 155 | 20 | 155 | 20 |
| 187 | 27 | 185 | 53 | 187 | 25 | 183 | 32 | 184 | 36 |
| 229 | 64 | 225 | 23 | 228 | 69 | 237 | 66 | - | - |
| - | - | - | - | - | - | - | - | 240 | 62 |
| - | - | 261 | 77 | 269 | 35 | 278 | 37 | 270 | 27 |
| 279 | 42 | - | - | 285 | 27 | 301 | 24 | 292 | 30 |
|  |  | 318 | 52 | - | - | - | - | - | - |
| 326 | 52 | - | - | 332 | 74 | 335 | 60 | 331 | 65 |
| - | - | - | - | 366 | 50 | - | - | - | - |
| 339 | 73 | 345 | 27 | - | - | - | - | - | - |
| 382 | 90 | 385 | 63 | - | - | 386 | 33 | 383 | 30 |
| 429 | 50 | 432 | 31 | 413 | 68 | 423 | 53 | 414 | 73 |
| 460 | 21 | 462 | 31 | 466 | 39 | 468 | 44 | - | - |
| - | - | - | - | - | - | - | - | 479 | 59 |
| 512 | 54 | 511 | 47 | 512 | 36 | 512 | 40 | 513 | 39 |
| 514 | 18 | 515 | 18 | 514 | 15 | 515 | 17 | 516 | 18 |
| - | - | - | - | 544 | 78 | - | - | - | - |
| - | - | - | - | - | - | 562 | 71 | 558 | 70 |
| 572 | 78 | 570 | 46 | - | - | - | - | - | - |
| - | - | - | - | 598 | 37 | - | - | 590 | 14 |
| - | - | 610 | 28 | - | - | 611 | 21 | 610 | 30 |
| 636 | 18 | - | - | 634 | 20 | 628 | 35 | 638 | 38 |
| - | - | 650 | 60 | - | - | - | - | - | - |
| 681 | 76 | - | - | 674 | 68 | 668 | 49 | 676 | 47 |
| 706 | 11 | 696 | 38 | 705 | 17 | 699 | 35 | 711 | 44 |
| 738 | 30 | - | - | 744 | 40 | 754 | 84 | - | - |
| - | - | 765 | 20 | 768 | 28 | 766 | 22 | 760 | 41 |
| - | - | - | - | - | - | - | - | 769 | 20 |
| 780 | 54 | 779 | 106 | 780 | 18 | 780 | 18 | 779 | 13 |
| - | - | 783 | 12 | 796 | 20 | 790 | 35 | 789 | 20 |
| - | - | - | - | 815 | 38 | - | - | 808 | 22 |
| 833 | 40 | - | - | 820 | 17 | 822 | 46 | 824 | 17 |
| - | - | - | - | 838 | 19 | 844 | 30 | 841 | 30 |
| 869 | 21 | 878 | 81 | 866 | 24 | 860 | 18 | 876 | 23 |
| 896 | 47 | - | - | 895 | 58 | 881 | 21 | 887 | 14 |
| 937 | 26 | - | - | 938 | 23 | 892 | 24 | 893 | 61 |
| - | - | - | - | - | - | 913 | 26 | - | - |
| - | - | - | - | - | - | 947 | 32 | 947 | 40 |
| *-* | *-* | *-* | *-* | *2935* | *201* | *-* | *-* | *-* | *-* |
| *3167* | *359* | *3056* | *280* | *3094* | *184* | *3048* | *259* | *-* | *-* |
| *-* | *-* | *3276* | *239* | *3239* | *148* | *3268* | *228* | *-* | *-* |
| *3421* | *326* | *-* | *-* | *3380* | *188* | *-* | *-* | *-* | *-* |
| 3517 | 78 | 3574 | 63 | 3582 | 128 | *-* | *-* | 3598 | 36 |
| 3609 | 92 | *-* | *-* | 3613 | 87 | 3609 | 91 | 3607 | 46 |
| *-* | *-* | *-* | *-* | *-* | *-* | *-* | *-* | 3644 | 15 |
| *-* | *-* | *-* | *-* | 3666 | 32 | 3660 | 44 | 3659 | 26 |
| *-* | *-* | *-* | *-* | 3679 | 20 | 3681 | 28 | 3679 | 15 |
| *-* | *-* | *-* | *-* | 3692 | 15 | 3692 | 13 | 3691 | *-* |
